# Role of Wnt canonical pathway in hematological malignancies

**DOI:** 10.1186/1756-8722-3-33

**Published:** 2010-09-15

**Authors:** Xueling GE, Xin Wang

**Affiliations:** 1 Department of Hematology, Provincial Hospital Affiliated to Shandong University, Jinan, Shandong, 250021, China

## Abstract

Wnt canonical signaling pathway plays a diverse role in embryonic development and maintenance of organs and tissues in adults. It has been observed that Wnt/β-catenin signaling pathway is involved in the pathogenesis of many carcinomas. Moreover, Wnt/β-catenin pathway has been revealed to be associated with angiogenesis. Wnt canonical pathway signaling has great potential as a therapeutic target. It has been disclosed that some hematological malignancies, such as chronic lymphocytic leukemia, mantle cell lymphoma, may occur partly due to the constitutive activation of Wnt canonical signaling pathway. This review will summarize the latest development in Wnt canonical signaling pathway and its roles in tumorigenesis and angiogenesis.

## Introduction

Wnt canonical signaling pathway acts a significant part in embryonic development and in maintenance of organs and tissues in adults. In the past two decades, medical scientists have devoted themselves to understanding the cellular and molecular mechanisms of Wnt signaling. A lot of studies indicate that Wnt canonical pathway involves in the pathogenesis of a range of disease including many kinds of carcinomas. Hematological malignancies are the types of carcinoma that affect blood, bone marrow and lymph nodes. They may derive from either of the two major blood cell lineages: myeloid and lymphoid cell lines. The incidence of hematological malignancies has been increasing steadily in the world for the past years, but their etiology and pathogenesis has not been well understood involving areas of chromosome aberrations, apoptosis inhibition, abnormal activation of signaling pathways, angiogenesis, et al. In this review, we focus on the role of Wnt canonical signaling in carcinomas, especially in hematological malignancies, and then disclose potential therapeutic opportunities of this pathway in hematological malignancies.

### Wnt canonical pathway

**Figure 1:**
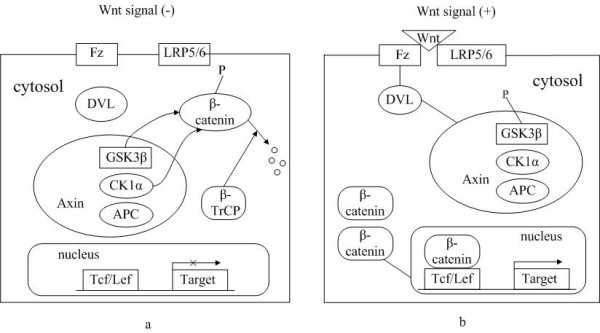
**Wnt canonical pathway**. (a) In the absence of a Wnt ligand, the cytoplasmic β-catenin is degraded by the "destruction complex". In this complex, Axin acts as an scaffold protein, which APC, GSK-3β and CK1α bind to facilitate the sequential phophorylation of β-catenin by kinase CK1α and GSK-3β. Accordingly, phosphorylated β-catenin is recognized by β-TrCP and constantly degraded by the ubiquitin-proteasome pathway. (b) Upon ligation of Wnts to their receptors composed of Fz proteins and LRP5/6, the cytoplasmic protein Dvl is recruited, phosphorylated and activated. Activation of Dvl induces the dissociation of GSK-3β from Axin and leads to the inhibition of GSK-3β. Next, the phosphorylation and degradation of β-catenin is inhibited as a result of the inactivation of the "destruction complex". Subsequently, stabilized β-catenin translocates into the nucleus. Nuclear β-catenin is the ultimate effector, binding to Tcf/Lef transcription factors to lead to changes in different target gene expressions.

Wnt signaling pathways are categorized as "canonical "and "non-canonical" Wnt pathways, which are β-catenin-dependent and β-catenin-independent signaling pathways, respectively. Here we will emphatically point out the role of Wnt canonical pathway in hematological malignancies. A simplified model of Wnt canonical pathway is delineated in Fig. 1. Wnts is a group of secreted cysteine-rich glycoproteins, which includes at least 19 identified members in diverse species ranging from round worm and insects to human[1]. In the absence of a Wnt ligand binding to its receptor complex, the cytoplasmic β-catenin is degraded by the "destruction complex". In this complex, Axin acts as an scaffold protein, which adenomatous polyposis coli (APC), glycogen synthase kinase 3β (GSK-3β) and casein kinase 1α (CK1α) bind to facilitate the sequential phophorylation of β-catenin in 45serine by kinase CK1α and 41'threonine, 37',33'serine by GSK-3β[2,3]. Accordingly, phosphorylated β-catenin is recognized by β-transducin-repeat-containing protein (β-TrCP) and constantly degraded by the ubiquitin-proteasome pathway. Wnt signaling is activated via ligation of Wnts to their respective dimeric cell surface receptors composed of the seven transmembrane frizzled (Fz) proteins and the low-density lipoprotein receptor-related protein 5/6 (LRP5/6). Upon ligation to their receptors, the cytoplasmic protein disheveled (Dvl) is recruited, phosphorylated and activated. Activation of Dvl induces the dissociation of GSK-3β from Axin and leads to the inhibition of GSK-3β. Next, the phosphorylation and degradation of β-catenin is inhibited as a result of the inactivation of the "destruction complex". Subsequently, stabilized β-catenin translocates into the nucleus. Nuclear β-catenin is the ultimate effector, binding to Tcf/Lef (T cell factor and lymphoid-enhancing factor) transcription factors that lead to changes in different target gene expressions that regulate cell proliferation, differentiation and survival, cell polarity and even angiogenesis.

### Role of Wnt canonical signaling in carcinomas

Wnt canonical signaling is involved in pathogenesis of several carcinomas and the mechanisms of its over-activation are varied. Dysregulation of Wnt/β-catenin signaling plays a central role in early events in colorectal carcinogenesis. The APC protein which acts as a tumor suppressor protein can down-regulate the transcriptional activation mediated by Wnt/β-catenin. Therefore, inactivation of APC tumor suppressor gene caused by mutation is related to the initiation of colorectal neoplasia and its protein products lose the function of down-regulation of Wnt signaling. Then, colorectal cancer occurs. Furthermore, mutations of β-catenin in the functionally significant phosphorylation sites have been detected in colorectal tumors[4]. In melanoma cell lines, abnormally high amounts and stabilization of β-catenin accompanied by mutations in β-catenin or alteration/missing of APC have been detected. Thus, genetic defects that result in up-regulation of β-catenin may play a role in melanoma progression[5]. Wnt canonical pathway has been confirmed to be related to initiation, development, progression and skeletal metastasis of prostate cancer in both human cancers and mouse models. It may result from mutation or altered expression of components of this pathway such as β-catenin and APC, which have been found in some types of prostate tumors and cancer cells[6,7]. Therefore, Wnt/β-catenin provides an attractive target for developing therapeutics of prostate cancers. Canonical Wnt pathway participates in many physiologic events in embryogenesis and is involved in embryogenic development of the ovary[8]. It also has an impact upon ovarian tumorigenesis especially a histologic subtype of epithelial ovarian cancer[9,10]. Non-small cell lung cancer(NSCLC) is one of the most common human carcinomas with a poor prognosis. Recent studies have revealed that the Wnt-1 overexpression, resulting in an aberrant and stabilized β-catenin expression, is associated with the expression of tumor-associated Wnt-targets(c-Myc, CyclinD1, Matrix Metalloproteinase 7), tumor proliferation, angiogenesis and a poor prognosis factor in NSCLC[11]. In addition, alterations of canonical Wnt signaling pathway due to frequent mutations in β-catenin have been detected in a wide range of other tumors, including hepatocellular carcinomas[12] and Wilms' tumors[13]. Mutations in the scaffold protein Axin [14] have been verified in some malignancies. Besides these mutations in intracellular signaling components, several tumors display a missing of expression of the secreted Wnt antagonists sFRPs and WIF1 resulting from silencing by promoter hypermethylation [15].

Since aberrant activation of Wnt canonical signaling pathway is diversely involved in pathogenesis of carcinomas, there has been great interest in developing therapeutics that circumvent it either by inhibiting Wnt mediated transcription or by inactivating the target genes. In some carcinomas, Wnt canonical signaling pathway has become the potential therapeutic target. The stabilized nuclear β-catenin due to the aberrant activation of Wnt signaling is an attractive therapeutic target for human cancers. Non-steroid anti-inflammatory drugs(NSAIDs) can suppress the activity of β-catenin. These drugs may inhibit Wnt/β-catenin signaling at multiple levels, including induction of β-catenin degradation[16] and disruption of the Tcf/β-catenin complex[17]. In addition, the transcriptional activity of Wnt/β-catenin can be inhibited by quercetin, a famous anti-tumor agent, in SW480 cell lines and also in HEK293 cells transiently transfected with constitutively active mutant β-catenin gene. The inhibitory mechanism is due to the decreased nuclear β-catenin and Tcf-4 proteins[18]. The way in which nuclear β-catenin and Tcf proteins were decreased needs to be further studied. The Wnt-Frizzled interaction can be antagonized by several secreted proteins, including Dickkopf1 (Dkk1), Wnt inhibitory factor 1(WIF-1) and secreted frizzled-related protein (sFRP) family members. Wnt/β-catenin pathway can become the potential therapeutic target of cancer achieved by expression of secreted antagonists of the pathway, such as Dkk1. In addition, small interference of RNA (siRNA) may eliminate components of Wnt/β-catenin signaling and can also be used to block this signaling. Others include small molecule inhibitors which can interfere the formation of the Tcf/β-catenin complex[19] or disturb the interaction of β-catenin with other co-activators[20] and monoclonal antibodies targeting the upstream signaling components such as Wnts ligands [21,22] or frizzled receptors, et al. The fact is that Wnt/β-catenin signaling pathway has great therapeutic potential in carcinomas.

### Canonical Wnt signaling and angiogenesis

Wnt signaling pathway has been observed to make a difference in vessel development and pathology and in survival and proliferation of primary endothelial cells. Several Wnt ligands have been demonstrated to be expressed in vascular endothelial cells in vitro, including Wnt-7a, Wnt-10b and in vascular smooth muscle cells including Wnt-5a[23]. In vivo, the fetal vessels of the placenta express Wnt-2[24] and the blood vessels of the mouse embryonic yolk sac express Wnt-5a and Wnt-10b[25]. Other components of this pathway such as Fz receptors have been demonstrated to be expressed in cultured endothelial cells and vascular smooth muscle cells[26,27]. During human embryonic development, nuclear and/or cytoplasmic β-catenin can be detected in placental villus capillaries, fetal capillaries, arteries and veins[28]. Furthermore, Wnt/β-catenin signaling may promote proliferation and survival in human endothelial cells via the induction of known angiogenic regulators; such as Interleukin-8 which is another transcriptional target of canonical Wnt pathway[29].

Angoigenesis is essential for tumor growth and metastasis. Studies have revealed the close relationship between canonical Wnt signaling pathway and angiogenesis of carcinomas. β-catenin accumulation has been involved in angiogenesis in brain cancer. β-catenin is found in the cytoplasm and nucleus of endothelium in neovessels of rat N-ethyl-N-nitrosurea-induced gliomas[30] and in the neovascular endothelial cells of medulloblastomas and other tumors of central nervous system[31]. However, accumulation of β-catenin in the cytoplasm or nucleus is rarely seen in cells of the normal adult brain vasculature[32]. A role for Wnt/β-catenin signaling in the vasculature is further supported by the identification of Wnt target genes that encode angiogenic regulators. Vascular endothelial growth factor A(VEGF-A) is a potent and widely distributed angiogenic peptide and has confirmed to be associated with the tumor angiogenesis and a poor prognosis[33,34]. It is also a target of canonical Wnt/β-catenin signaling pathway[35]. Seven β-catenin/Tcf binding sites occur in the VEGF-A promoter[36]. A recent study on NSCLC has disclosed that the Wnt1 expression correlates with the intratumoral VEGF-A expression with the action of elevating the activity of Wnt/β-catenin pathway[10]. In the meantime, a significant proportion of human colorectal cancers have an activating mutation in Wnt/β-catenin pathway resulting in the abnormal expression of VEGF[37].

It is believed that Wnt signaling pathway is vital for tumor neovascularization and is a great potential in blocking tumor invasion and metastasis. To further confirm the role of Wnt/β-catenin signaling pathway in tumor angiogenesis and growth, Wnt antagonists WIF1-Fc and sFRP1-Fc were used to treat hepatocellular carcinoma tumors. They revealed that these two fusion proteins could inhibit Wnt signaling and exerted potent antineoplastic activity by increasing apoptosis of tumor cells and by impairing tumor vascularization; including reducing the microvessel density, decreasing expression of vascular endothelial growth factor and stromal cell-derived factor-1[38].

### Role of Wnt canonical pathway in hematological malignancies

Hematopoiesis is a continuous process in which stem/progenitor cells develop into mature blood cellular components. Wnt/β-catenin signaling pathway has been shown to have an effect on controlling the proliferation, survival and differentiation of hematopoietic cells[39]. The gene products of the Wnt family, functioning as hematopoietic growth factors, may exhibit higher specificity for earlier progenitor cells[40]. Wnts have additionally been shown to participate in hematopoiesis in which Wnt-11 induced bone marrow cells to develop into a variety of different lymphoid cell types[41]. Wnt3a signaling not only provides proliferative stimuli such as for immature thymocytes, but also regulates cell fate decisions of HSC during hematopoiesis[42]. More recently, gain of function studies have demonstrated that constitutively activated β-catenin in hematopoietic stem cells blocks multilineage differentiation; including B cell differentiation at early stages, suggesting the importance of fine tuning of Wnt/β-catenin signaling pathway for normal B cell development and function [43,44]. Frizzled 9 knockout in mice leads to abnormal B-cell development [45]. Wnt signaling is required for thymocyte development[46] and plays a key role in the maintenance of stemness in mature memory CD8+T cells[47]. Constitutive activation of β-catenin promotes the expansion of multipotential HSCs [44,48]. However, the influence of Wnt/β-catenin pathway on mature B cells is not obvious because they do not express TCF/LEF factors[43]. Excessive stimulation of the Wnt cascade may lead to transformation of HSCs[44,48] and is noticeable in the neoplasms of myeloid and lymphoid lineages. Thus any aberrant signaling through this pathway may have a negative influence on hematopoiesis and may involve in lymphomagenesis.

Aberration of Wnt pathway and the related proteins are detected in many hematological patients[49]. Activation of Wnt signaling pathway has been implicated in the pathogenesis of leukemia. More recently, β-catenin activation coupled with GSK3β inactivation, has been demonstrated in chronic myeloid leukemia(CML) in blast crisis and precursor B-cell acute lymphoblastic leukemia(ALL)[50]. The function of canonical Wnt pathway is epigenetically regulated by methylation of Wnt antagonists and has prognostic relevance in acute myeloid leukemia(AML)[51]. Secreted Frizzled-related protein genes (sFRPs), functioning as Wnt signalling antagonists, have been found to be downregulated or inactivated by promoter hypermethylation in ALL and AML[52]. In addition, small molecule inhibitors of Wnt signaling effectively induce apoptosis in AML cells. Consequently, targeting this pathway seems to be an innovative approach in the treatment of AML[53]. Studies have demonstrated that deregulation of Wnt signaling pathway plays a role in the pathogenesis of CML. However, β-catenin amino-terminal mutations are not observed or are very rare and therefore are not the underlying mechanism of activated Wnt signaling in CML[54]. There must be other mechanisms for deregulating canonical Wnt signaling in CML. Wnt signaling genes are also overexpressed and may be pathologically reactivated in other neoplastic transformation of mature B cells, such as chronic lymphocytic leukemia (B-CLL). Uncontrolled Wnt signaling may contribute to defects in apoptosis that characterizes this malignancy[55,56].

Epstein-Barr Virus (EBV) is consistently detected in the endemic form of Burkitt's lymphoma (BL). An increase in both free and total β-catenin was seen in EBV-infected BL cells compared to EBV-negative cells[57]. The involvement of Wnt/β-catenin pathway in cell-cycle regulation, proliferation and invasion contributing to enhanced proliferative and metastatic properties of multiple myeloma (MM), were documented[58]. Furthermore, β-catenin small interfering RNA treatment inhibited the growth of multiple myeloma tumors in a xenograft model. As a result, β-catenin is the attractive novel target for treating multiple myeloma and other hematologic malignancies with aberrant canonical Wnt signaling[59]. Aberration of Wnt canonical pathway (WCP) may exist in mantle cell lymphoma(MCL) and appears to promote tumorigenesis in MCL. MCL tumors and cell lines highly and consistently expressed Wnt3 and Wnt10. Then, β-catenin was localized to the nucleus and transcriptionally active in MCL cell lines examined and more than half of the MCL tumors showed nuclear localization of β-catenin by immunohistochemistry, which obviously correlated with the expression of the phosphorylated/inactive form of GSK-3β(pGSK-3β)[60]. Of the clinical parameters, continuous pGSK-3β status had a significant correlation with absolute lymphocyte count in blood and negative pGSK-3β expression was significantly correlated with a longer overall survival in MCL[61]. Frequent β-catenin overexpression and accumulation may play an important part in the development of cutaneous lymphomas and it's mechanisms may not be associated with exon 3 mutation but others[62]. Nuclear localization of β-catenin was detected in extranodal marginal zone lymphoma by immunohistochemistry[63]. Scientists have revealed that esearchethacrynic acid (EA) and the antifungal agent ciclopiroxolamine (cic) could inhibit Wnt/β-catenin signalling in the myeloma cell line OPM-2 and three lymphoma cell lines (OCI-LY8-LAM-53, SU-DHL-4 and Raji) in vitro and led to apoptosis and a significant decrease of viability in lymphoma and its cell lines[64]. The Hedgehog (Hh) inhibitor, cyclopamine, and the Wnt inhibitor, quercetin, could suppress the growth of a number of leukemia and lymphoma cells[65]. Therefore, there is great potential that Wnt/β-catenin pathway can act as a therapeutic target of lymphoma and myeloma.

## Conclusion and future directions

Wnt canonical signaling pathway is not only involved in cell survival, differentiation, apoptosis and maintenance of homeostasis, but also related to the pathogenesis of many carcinomas and hematological malignancies. Moreover, Wnt/β-catenin pathway has been revealed to be associated with angiogenesis of tumors. Its aberration has been detected in leukemia, myeloma and lymphoma. Canonical Wnt signaling may act as a potentially useful therapeutic target for hematological malignancies. Ultimately, further investigation is needed to interfere with Wnt signaling which may lead to new anti-cancer therapies.

## Competing interests

The authors declare that they have no competing interests.

## Authors' contributions

Both authors participated in drafting and editing the manuscript. Both authors read and approved the final manuscript.
